# EXPLORING MEDICATION CONVERSATIONS IN NURSING HOME CARE CONFERENCES: A QUALITATIVE STUDY

**DOI:** 10.1093/geroni/igae098.3461

**Published:** 2024-12-31

**Authors:** Te-Lien Ku, Jingxi Li, James Ford, Tonya Roberts

**Affiliations:** University of Wisconsin Madison, Madison, Wisconsin, United States; University of Wisconsin Madison, Madison, Wisconsin, United States; University of Wisconsin Madison, Madison, Wisconsin, United States; University of Wisconsin - Madison, Madison, Wisconsin, United States

## Abstract

Care conferences (CCs) represent a structured opportunity for nursing home (NH) staff, residents, and families in collaborative discussions about medication care planning, a fundamental component ensuring resident disease management and safety. Yet, how medication issues are addressed within CCs remains unexplored. This study aimed to describe how medications were discussed and who discussed them through a convenience sample of naturally observed CCs (n=15) in a 40-bed, nonprofit, short-stay, Midwestern NH. Observations were audio-recorded, transcribed verbatim, and analyzed using thematic analysis. CCs lasted 20 to 55 minutes each (mean=36 minutes). The number of attendees in CCs ranged from 5 to 15, with 6 being the most frequent (33.3%). Of these, 14 CCs (93%) involved medication conversations. A variety of staff attended CCs; however pharmacists were absent. Social workers and registered nurses were primarily engaged in medication conversations. Residents and families typically participated passively. Medication conversations centered around three main themes: (1) Information Seeking and Sharing—unifying participants’ understanding regarding current medication and offering opportunities for consultation; (2) Decision Making—negotiating and making decisions about adding, changing, or stopping the resident’s medication during NH stay; and (3) Discharge Planning—confirming the continuity of the resident’s medication post-discharge and navigating access to managing medication resources. The findings of this study could enhance our understanding of how medications-related discussions are conducted during CCs in NHs. Future studies can further identify the different professional roles of NH staff in medication care planning according to residents’ and families’ needs, and explore the inclusion of pharmacists in CC.

